# Local Tumor Recurrence and Escape from Suppression of Bone Resorption With Denosumab Treatment in Two Adolescents With Giant Cell Tumors of Bone

**DOI:** 10.1002/jbm4.10196

**Published:** 2019-06-18

**Authors:** Umar Akel, Marie‐Eve Robinson, Joel Werier, Raja Rampersaud, Kawan Rakhra, Donna Johnston, Victor N Konji, Jinhui Ma, Marika Pagé, Mary Ann Matzinger, Leanne M Ward

**Affiliations:** ^1^ Pediatric Bone Health Clinical Research Program, Children's Hospital of Eastern Ontario Research Institute Ottawa Canada; ^2^ Department of Pediatrics Division of Endocrinology, Children's Hospital of Eastern Ontario Ottawa Canada; ^3^ Department of Surgery Division of Orthopaedic Surgery, The Ottawa Hospital Ottawa Canada; ^4^ Department of Surgery Division of Orthopaedics, University Health Network Toronto Canada; ^5^ Department of Pediatrics Division of Hematology/Oncology, Children's Hospital of Eastern Ontario Ottawa Canada; ^6^ Department of Health Research Methods Evidence, and Impact, McMaster University Hamilton Canada; ^7^ Department of Diagnostic Imaging Children's Hospital of Eastern Ontario Ottawa Canada

**Keywords:** DENOSUMAB, GIANT CELL TUMOR, ADOLESCENTS, HYPOCALCEMIA, HYPERCALCEMIA, REBOUND

## Abstract

Giant cell tumors of bone (GCTB) may be difficult to resect because of size or location. We describe two adolescents who were treated with denosumab and followed for tumoral and biochemical responses. Denosumab was effective in achieving sufficient regression to allow surgical resection and in preserving peritumor cortical bone. Reactivation of bone resorption markers was noted while the patients were receiving monthly denosumab. One patient suffered a local tumor recurrence. Denosumab was safe in enabling surgical resection of GCTB. However, the effect was transient, with an escape of resorption markers and tumor recurrence. © 2019 The Authors. *JBMR Plus* published by Wiley Periodicals, Inc. on behalf of American Society for Bone and Mineral Research.

## Introduction

Giant cell tumors of bone (GCTB) are rare, locally aggressive tumors composed of macrophages, multinucleated osteoclast‐like giant cells and mononuclear stromal cells.^1^ Surgical removal is the typical first‐line treatment; however, resection may be technically challenging depending on the location and size of the tumor, and the recurrence rate is high (15% to 52.9%).^2^, ^3^, ^4^, ^5^ Receptor activator of NF‐κB ligand (RANKL) and its receptor, RANK, are expressed by stromal cells and osteoclast‐like giant cells within GCTB. They play a role in tumor viability and are principally responsible for the osteolytic behavior of GCTB by promoting skeletal resorption through stimulatory effects on osteoclast formation, differentiation, and proliferation.^6^ Denosumab is a human monoclonal antibody that inhibits RANKL and prevents skeletal resorption. It is therefore an attractive option in the GCTB setting, in order to reduce the size of the tumor and to preserve the integrity of adjacent bone tissue.^1^

The purpose of this report was to describe the clinical course of GCTB in two teenage boys, both with surgically challenging tumors that led to use of denosumab to reduce tumor size, reconstitute adjacent bone tissue, and facilitate subsequent surgical intervention. We also describe the bone and mineral ion changes in response to denosumab, given its potent antiresorptive action on the skeleton.

## Case Report

Two boys with GCTB were referred to the Pediatric Bone Health Clinic at the Children's Hospital of Eastern Ontario for consultation on the use of denosumab in the setting of surgically challenging GCTB. Subcutaneous denosumab was administered at doses of 120 mg weekly for 3 weeks, followed by monthly dosing of 120 mg for 8 months in patient 1 and for 11 months in patient 2.^7^ Neither patient was treated with a bisphosphonate in the past. To mitigate denosumab‐induced hypocalcemia, both patients received cholecalciferol 3000 IU daily, calcium carbonate 600 mg per day, and Rocaltrol 0.25 mcg twice daily. Rocaltrol was discontinued 5 days after patient 1 received his fourth denosumab dose. Both patients were followed for at least 8 months after discontinuation of denosumab and underwent bone biochemical profiling, including parathyroid hormone (PTH), ionized calcium, serum phosphate, 25‐hydroxyvitamin D, alkaline phosphatase (ALP), and C‐telopeptide of type 1 collage (CTX). Informed consent was obtained as per local Institutional Review Board requirements.

### Patient 1

**Figure 1:**
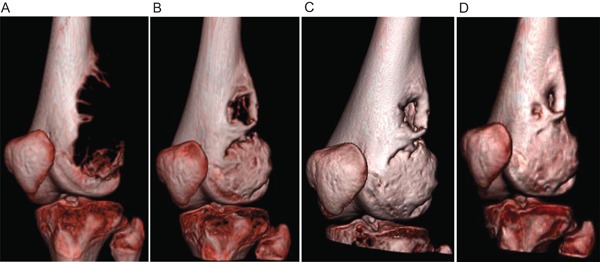
Computed tomography showing regression of the giant cell tumor of bone in patient 1. (*A*) before denosumab initiation, (*B*) 1.5 months after denosumab initiation, (*C*) 3 months after denosumab initiation, and (*D*) 8 months after denosumab discontinuation.

A 16‐year‐old boy presented with a rapidly growing, painful GCTB of the left distal femur. The volume of the tumor was 503.7 cm^3^ at consultation. After 8 months on denosumab therapy, computerized tomography demonstrated tumor regression to 94.8 cm^3^, with reconstitution of the deficient adjacent bone cortices (Fig. 1). He then underwent surgical resection with extended intralesional curettage technique and bone allograft reconstruction. There was complete resolution of pain and recovery of mobility postoperatively. A slow recurrence was noted 14 months after denosumab cessation; therefore, a second successful intralesional curettage operation was completed 1 year after the index surgery.

**Figure 2:**
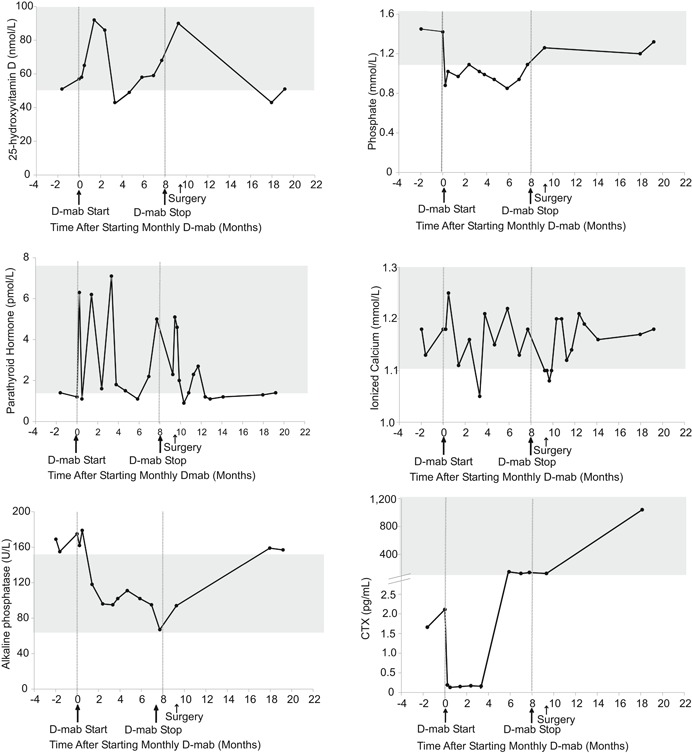
Serum biochemical response to denosumab in patient 1. Normal ranges are shown in gray. D‐mab = denosumab.

Mild hypocalcemia was observed after 3.5 months of denosumab treatment (ionized calcium 4.20 mg/dL, N: 4.4 to 5.2), while the patient was nonadherent to cholecalciferol supplementation. This was treated by increasing cholecalciferol to 4000 IU/d, by increasing calcium carbonate to 1200 mg, and by restarting calcitriol 0.25 mcg twice daily. On the other hand, rebound hypercalcemia was not observed up to 11.5 months after denosumab discontinuation, while mild asymptomatic hypophosphatemia was noted throughout treatment. Serum CTX rebounded above baseline values at 3.5 months while on monthly denosumab and continued to climb post‐treatment discontinuation (Fig. 2).

### Patient 2

A 17‐year‐old boy presented with a recurrence of tumor after index resection of a GCTB involving the T_5_ vertebral body and large soft tissue infiltration. Back pain started 3 months before diagnosis, and he complained of dyspnea on exertion and cough due to pulmonary compression, and left leg and foot numbness due to spinal cord impingement. Within 72 hours of denosumab initiation, pulmonary and spinal cord compression symptoms resolved and back pain improved. The tumor volume decreased from 37.6 cm^3^ to 8.7 cm^3^ after 11 months on denosumab, facilitating complete resection with negative resection margins. He did not have a tumor recurrence up to 3 years later.

**Figure 3:**
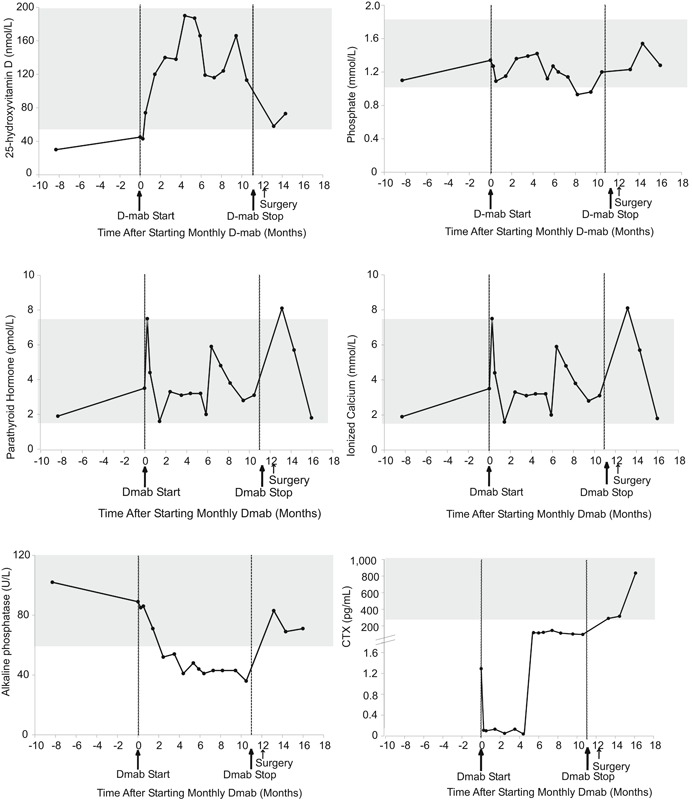
Serum biochemical response to denosumab in patient 2. Normal ranges are shown in gray. D‐mab = denosumab.

Calcium levels remained within the acceptable range throughout and after treatment. Mild hypophosphatemia was observed at 8 months post‐treatment initiation and resolved at 10.5 months without specific intervention apart from encouraging dairy intake through diet. The serum biochemical response is depicted in Fig. 3.

## Discussion

Denosumab 120 mg/wk for 3 weeks followed by 120 mg/mo was a safe and effective approach in achieving GCTB regression, adjacent bone reconstitution, and symptom control in two adolescents, allowing for successful surgical removal. Similar positive findings have been described in the literature in some, but not all, teenagers and adults. Rekhi and colleagues reported that denosumab followed by surgical resection resulted in complete eradication of giant cells on histopathologic examination in 55% of patients (ages 16 to 47 years).^1^ Similarly, Branstetter and colleagues used denosumab to treat inoperable tumors in 20 adults, resulting in decreased osteoclast‐like giant cells in 90% of cases.^8^ Another study by Müller and colleagues showed that among 25 patients (ages 15 to 72 years) treated before and/or after surgery with denosumab, 40% could undergo surgical downstaging.^9^ Finally, the interim report of a larger safety and efficacy phase 2 trial using denosumab in 282 adults and skeletally mature adolescents showed that 62% of patients treated before their surgery had a less morbid procedure than planned,^7^ while pain reduction was achieved in 28% to 50% of cases.

Although denosumab was successful in facilitating surgical resection in our series, our patient with the distal femur GCTB had a local tumor recurrence necessitating a second surgery. Recently, concern has been raised that denosumab may even increase the risk of local recurrence in patients with GCTB of the extremities, specifically after curettage.^10^ Errani and colleagues reported in a retrospective review that 60% of patients with GCTB of the extremities who had been treated with denosumab and curettage had a local recurrence, compared with 16% of patients treated with curettage alone.^10^ In addition, 80% of patients treated with denosumab and curettage achieved joint preservation, compared with 94% of patients who underwent curettage alone. Denosumab was the only independent factor associated with an inferior prognosis for recurrence‐free survival and joint preservation after a median of 7 years’ follow‐up.^10^

In addition to the observations of Errani and colleagues,^10^ Traub and colleagues reported that the new osseous tumor matrix and thickened cortical bone that developed after denosumab treatment raised new surgical challenges by impairing the surgeon's ability to delineate the true extent of the tumor.^11^ These authors theorized that tumor cells might be better able to hide within the thickened cortex and subchondral bone tissue, which might in turn increase the frequency of local recurrence.^11^ Of relevance to these tumoral considerations, we observed an escape from suppressed bone resorption, with a precipitous rise to above baseline in serum CTX by 5 months post‐denosumab initiation, while receiving monthly denosumab dosing. The notion of “rebound effect” is emerging for a number of clinical outcomes in the denosumab treatment setting, including rebound vertebral fractures and loss of bone mineral density after denosumab discontinuation in women with postmenopausal osteoporosis.^12^ Rebound hypercalcemia in young children with osteogenesis imperfecta while on active therapy^13^, ^14^, ^15^ and in two adolescents and one young adult with GCTB have also been described. With respect to side effects, although not observed in our patients, Uday and colleagues reported one adolescent with denosumab‐treated GCTB who experienced osteonecrosis of the jaw.^15^ This adolescent received a significantly larger cumulative dose of denosumab than our patients (5520 mg compared with an average cumulative dose of 1500 mg in our patients).

In summary, denosumab was effective for GCTB regression, facilitating surgical resection, and in alleviating symptoms before resection. Neither hypo‐ nor rebound hypercalcemia were observed with our calcium and vitamin D management protocol. On the other hand, the local recurrence of a distal femur GCTB after curettage, combined with recent observations in the literature that denosumab is associated with an increased risk of recurrence after this surgical technique,^10^ raises the need for further studies to understand the benefits and risks of denosumab in the GCTB setting.

## Disclosures

LMW has been a consultant to and is participating in clinical trials with Amgen. All other authors state that they have no conflicts of interest.
